# Association between VExUS score and worsening renal function during diuretic therapy in the ICU

**DOI:** 10.1186/s40635-026-00890-9

**Published:** 2026-03-31

**Authors:** Corentin Evezard, Pierre Alain Bahr, Maxime Nguyen, Belaid Bouhemad, Pierre-Gregoire Guinot

**Affiliations:** 1 Department of Anaesthesiology and Critical Care Medicine, Dijon University Medical Centre, 21000 Dijon, France; 2 University of Burgundy and Franche-Comté, LNC UMR1231, 21000 Dijon, France; 3 Center for Translational and Molecular Medicine (CTM), INSERM UMR1231, Lipness Team, Dijon, France

## Abstract

**Background:**

In intensive care unit (ICU) settings, venous excess ultrasound (VExUS) score has gained attention for predicting Acute Kidney Injury (AKI). This led to the identification that venous congestion via VExUS should prompt diuretic therapy. However, in acute heart failure (AHF), a share of the literature considers creatinine elevation as a sign of efficient decongestion. Thus, the relationship between VExUS, diuretic response, and renal outcomes remains unclear in ICU patients.

**Methods:**

Secondary analysis of a prospective observational study conducted in a cardiovascular ICU (2019–2022). Adult patients with clinical signs of fluid overload receiving loop diuretic treatment were included. Patients were divided into two groups based on their highest VExUS score severity over 24 h: congestive (VExUS ≥ 2) versus non-congestive (VExUS < 2). The primary outcome was WRF at ICU discharge. Secondary outcomes included diuretic response parameters, hemoconcentration, and distinction between "pseudo-WRF" (WRF with hemoconcentration) and "true-WRF" (WRF without hemoconcentration).

**Results:**

Seventy-seven patients were analyzed (37 with VExUS < 2, 40 with VExUS ≥ 2). WRF occurred in 14 patients (37.8%) in the non-congestive group versus 8 patients (20.0%) in the congestive group (p = 0.139). No significant differences were observed between groups for diuretic response parameters: loop diuretic-adjusted diuresis at 2 h (545 vs 600 mL/40 mg, p = 0.950), natriuresis (104 vs 93.0 mmol/L, p = 0.355), cumulative fluid removal (−685 vs −1141 mL, p = 0.895), or cumulative loop diuretic prescription (120 vs 100 mg, p = 0.303). Hemoconcentration rates were similar between groups (48.6% vs 32.5%, p = 0.226), as were pseudo-WRF rates (16.2% vs 7.5%, p = 0.241).

**Conclusions:**

In critically ill patients systematically treated with loop diuretics, VExUS score was not significantly associated with worsening renal function or diuretic response parameters. These preliminary findings suggest that larger studies may be needed to better understand the potential relationship between VExUS and renal outcomes in this patient population.

**Supplementary Information:**

The online version contains supplementary material available at 10.1186/s40635-026-00890-9.

## Introduction

In critically ill patients, persistent positive fluid balance and venous congestion are associated with poor outcomes, particularly acute kidney injury (AKI) and increased mortality [1, 2]. The VExUS (Venous Excess Ultrasound) score, which combines multiple venous ultrasound criteria across three territories (hepatic, portal, and renal) with inferior vena cava measurements, is described as a non-invasive tool for assessing this venous congestion and has demonstrated good correlation with AKI occurrence [3–5]. Therefore, VExUS monitoring could potentially help in selecting patients suffering from congestive state, for whom decongestive therapy would be relevant.

However, most studies regarding VExUS were not conducted in the context of systematic diuretic administration. Therefore, the relevance of VExUS for predicting diuretic response and subsequent renal course is still questionable, while diuretic response is key to determining renal outcome: The Furosemide Stress Test (FST), which consists in a standardized loop diuretic prescription, has demonstrated that low diuresis and natriuresis following loop diuretic administration strongly correlate to AKI occurrence [6–8].

In addition, in acute heart failure (AHF), authors have emphasized that some magnitude of worsening renal function (WRF) might be associated with better outcomes [9]. Efficient decongestion might induce some plasmatic concentration, resulting in creatinine increase that meets WRF definition. This creates uncertainty about expected results when high VExUS scores trigger decongestive therapy, since WRF could be interpreted as either beneficial or detrimental. Various definitions of hemoconcentration have been used to explore decongestion through hematocrit, serum protein and albumin concentration [10]. Despite being associated with WRF, hemoconcentration has been linked to better prognosis in AHF patients, indicating decongestion efficiency, and supporting this concept of “pseudo-WRF” [11].

We conducted a secondary analysis of a prospective observational study that utilized ultrasound measurements in patients with congestive conditions who were treated with loop diuretics [12]. Our primary objective was to assess the association of the VExUS score and the usual definition of WRF at the time of ICU discharge. We also aimed to evaluate the association of congestive VExUS score with diuretic response, and pseudo-WRF as defined with hemoconcentration markers.

## Material and methods

### Patients

We performed a secondary analysis of a prospective, observational, single center study in a cardiovascular medico-surgical ICU of a tertiary university medical center (Dijon, France) between 2019 and 2022 [12]. The research was approved by the institutional review board. Patients or their next of kin have received a written informed letter and gave consent to participate. The study was performed following the ethical standards laid down in the 1964 Declaration of Helsinki. Inclusion criteria were: adults (≥ 18 years old) for whom the clinician introduced loop diuretic treatment, clinical signs of fluid overload (pulmonary crackle, peripheral edema, jugular vein turgor, or hepatojugular reflux), no fluid-responsiveness after passive leg-raising, first loop diuretic treatment, and natriuresis measurement. Non-inclusion criteria were prior diuretic treatment during ICU stay and permanent atrial fibrillation, dialysis.

### Echocardiographic measurements

An experienced physician (P.A.B.) performed all transthoracic echocardiography and venous Doppler examinations using a Philips Affinity ultrasound system. Measurements were averaged over five cardiac cycles. Data were acquired and stored for later analysis. The images were reviewed offline by an experienced operator blinded to the study outcomes. The attending physician was unaware of the results of the ultrasound examination. Cardiac measurements followed current guidelines [13], while venous Doppler assessments were performed as previously described (Supplementary Table 1) [12, 14]*.* The electrocardiogram was recorded during the ultrasound loop to ensure accuracy in Doppler measurements. These measurements allowed to calculate the VEXUS score (grading system C) as previously described (Supplementary Table 2) [14]. Intra-rater variability of each ultrasound marker was evaluated by calculating the maximum absolute difference between measurements divided by the mean of all observations (Supplementary Table 3) [15].

### Data collection and study protocol

Data collection included demographics, clinical, biological, and ultrasound monitoring. Demographic features included age, gender, size, weight, Body Mass Index (BMI), comorbidities, SAPS II (Simplified Acute Physiologic Score II), and medical/surgical admission. Clinical features included mean arterial pressure (MAP), central venous pressure (CVP) retrieved on a central line, heart rate, catecholamine infusion rate, oxygen saturation, diuresis and 24-h hydric balance. Biologic monitoring involved hemoglobin, hematocrit, protein levels, albumin levels, N-terminal Pro-Brain Natriuretic Peptide (NT-pro-BNP), creatinine, and natriuresis.

Loop diuretic prescription was left at the discretion of the clinician involved in the patient’s care. Diuresis and natriuresis were then assessed 2 h later [9]. Ultrasound and echocardiography were realized at inclusion, before diuretic administration, at 2 h and at 24 h. Biologic monitoring was retrieved at inclusion and at 24 h. Creatinine, diuresis, cumulative fluid balance, and total diuretic administration were monitored over 48 h.

### Definitions and outcomes

AKI was defined according to the KDIGO classification [16]. WRF was defined by creatinine elevation over 26.5 µmol/L within 48 h after admission or a relative 50% increase between baseline and ICU discharge [16, 17].

Response to diuretic was assessed using natriuresis and diuresis 2 h after diuretic administration [9]. Because diuretic prescriptions were not standardized according to the FST method, diuresis was adjusted based on the diuretic dosage (milliliters of diuresis per 40 mg of loop diuretic, or ml/40 mg). Furosemide was the only loop diuretic used in the study.

Patients were divided according to the severity of congestion, assessed by the maximum VExUS score during the first 24 h. Patients were classified as “congestive” when VExUS score was equal or above 2, and “non-congestive” when VExUS score was lower than 2 [3].

Since there is no universally accepted definition, hemoconcentration was defined as an increase in at least two of the following criteria between baseline and day 1: hematocrit, protein, and albumin [10, 11]. We define “pseudo-WRF” as the occurrence of WRF in the presence of hemoconcentration, while “true-WRF” was defined as WRF occurring in the absence of hemoconcentration.

The primary outcome was the association between the VExUS score measured at the time of diuretic administration and WRF at ICU discharge. The secondary outcomes were the association between diuretic efficiency, defined as diuresis normalized to the first dose of loop diuretic (ml/40 mg of loop diuretic) [18], and natriuresis 2 h after diuretic administration, distinction between “pseudo-WRF” and “true-WRF”, VExUS score at 2 h and 24 h, fluid balance at discharge, cumulative loop diuretic dosage over ICU stay, and mortality rate during ICU stay.

### Statistics

Patients were categorized into two distinct groups based on the highest VExUS score recorded within the first 24 h following admission: non congestive patients and congestive patients. Quantitative data are presented as median (interquartile range) or mean (standard deviation). Qualitative data are presented as frequencies and percentages. Normality was assessed using the Shapiro–Wilk test and histograms. For quantitative variables, comparisons between groups used the Mann–Whitney test or Student's t-test, as appropriate. Qualitative variables were compared using Chi-square or Fisher's exact test when expected cell frequencies were ≤ 5. When an ultrasound marker was missing, VExUS was classified using available data when possible; otherwise, the last known value was carried forward, if necessary, to enable classification, following the methodology of Souligny et al. [3]. Other missing data patterns are reported in Supplementary Table 4. Sensitivity analyses were performed to ensure that hemoconcentration markers were not confounded by concomitant fluid resuscitation. Analyses were restricted to the subset of patients achieving a negative cumulative fluid balance. Multivariable analysis was performed using Firth's penalized logistic regression to adjust for baseline variables that differed significantly between groups. This method was selected given the limited events-per-variable ratio. Statistical analyses were performed using R software version 4.2.2 (2022-10-31 ucrt), with dedicated packages. Statistical significance was set as p < 0.05.

## Results

### Population characteristic

Seventy-seven patients were included in the analysis. Demographic data are displayed in Table 1. The median age was 69 years old [62; 75] and 62% of the population were men. The main comorbidities were high blood pressure (n = 50, 65%), ischemic heart disease (n = 35, 45%), diabetes (n = 26, 34%), and prior stroke (16%, n = 12). Nineteen patients (25%) were receiving norepinephrine infusion at inclusion. The median loop diuretic dose administered at inclusion was 40 mg [40; 65]. The median serum creatinine at baseline was 83 µmol/L [55; 121].

**Table 1:** Patient characteristics at baseline

	Whole population (n = 77)	VExUS < 2 (n = 37)	VExUS ≥ 2 (n = 40)	*p*
Demographic data				
Age (years)	69 [62; 75]	71 [64; 75]	67 [61; 74]	0.382
Sex (male), n (%)	48 (62%)	20 (54.1%)	28 (70.0%)	0.227
SAPSII	46 [33; 58]	44 [35; 56]	46 [28; 58]	0.916
BMI (kg/m^2^)	26.4 [23.9; 30.1]	26.9 [23.9; 31.3]	26.1 [24.1; 29.7]	0.652
Medical history				
Atrial fibrillation, n (%)	8 (10%)	4 (10.8%)	4 (10.0%)	1.000
Diabetes mellitus, n (%)	26 (34%)	14 (37.8%)	12 (30.0%)	0.627
HBP, n (%)	50 (65%)	25 (67.6%)	25 (62.5%)	0.821
Arteritis, n (%)	6 (8%)	3 (8.11%)	3 (7.50%)	1.000
Stroke, n (%)	12 (16%)	3 (8.11%)	9 (22.5%)	0.154
CAD, n (%)	35 (45%)	15 (40.5%)	20 (50.0%)	0.546
Surgical admission, n (%)	56 (73%)	22 (59.5%)	34 (85.0%)	0.024
Clinical and biological data at admission				
Cardiac index (L/min/m^2^)	2.35 [1.98; 2.97]	2.61 [1.98; 3.00]	2.50 [1.99; 2.95]	0.574
MAP (mmHg)	85 [72; 92]	89 [75; 93]	80 [71; 89]	0.060
Norepinephrine, n (%)	19 (25%)	8 (21.6%)	11 (27.5%)	0.739
CVP (mmHg)	14 [10; 16]	14 [9; 16]	14 [12; 16]	0.130
Loop diuretic (mg)	40 [40; 80]	40 [20; 80]	40 [40; 65]	0.608
KDIGO, n (%)				0.669
0	36 (47%)	19 (51.4%)	17 (42.5%)	
1	30 (39%)	12 (32.4%)	18 (45.0%)	
2	8 (10%)	4 (10.8%)	4 (10.0%)	
3	3 (4%)	2 (5.41%)	1 (2.50%)	
Creatinine (µmol/L)	83.0 [55.0; 121.0]	83.0 [52.0; 131]	83.0 [60.2; 106]	0.858
NT-pro-BNP (pg/mL)	3004 [929; 5821]	2489 [693; 5134]	3540 [1468; 6818]	0.147
Hemoglobin (g/dL)	10.0 [9.0; 11.6]	10.1 [8.9; 11.3]	9.8 [9.1; 11.8]	0.927
Hematocrit (%)	30.5 [27.5; 34.3]	30.6 [27.5; 34.3]	30.0 [27.4; 33.9]	0.668
Protein (g/L)	57.0 [54.0; 62.0]	57.0 [55.0; 63.0]	57.5 [53.0; 62.0]	0.465
Albumin (g/L)	24.0 [21.0; 27.0]	23.0 [20.0; 27.0]	24.5 [21.0; 27.3]	0.414

Data are presented in mean (standard deviation), median [25th–75th percentile] or frequencies (%). *HBP* High Blood Pressure, *CAD* Coronary Artery Disease

Forty (52%) patients were classified as congestive and 37 (48%) as non-congestive. Congestive patients had a higher proportion of surgical admissions (85.0% vs 59.5%, p = 0.024). No significant differences were observed between groups regarding other baseline characteristics. Notably, NT-pro-BNP did not differ significantly between groups, no difference could be identified among hemoconcentration markers. The median length of stay was 5 days [3–10]. Additionally, no patient received a blood transfusion or exogenous protein administration during the study period.

### Primary outcome

Worsening renal function at discharge occurred in 14 non-congestive patients (37.8%) compared to 8 congestive patients (20.0%). The difference was not significant (*p* = 0.139). Results are shown in Table 2*.*

**Table 2:** Outcomes according to the highest VExUS score within 24 h

	VEXUS < 2 (n = 37)	VEXUS ≥ 2 (n = 40)	*p*
Primary outcome			
WRF, n (%)	14 (37.8%)	8 (20.0%)	0.139
Secondary outcomes			
Loop diuretic adjusted diuresis at 2 h (ml/40 mg of loop diuretic)	545 [251; 1038]	600 [334; 858]	0.950
Natriuresis at 2 h (mmol/L)	104.0 [68.5; 122.0]	93.0 [71.5; 111.0]	0.355
Cumulative Loop Diuretic prescription (mg)	120 [67; 161]	100 [45; 140]	0.303
Cumulative Fluid Removal (mL)	−685 [−2418; −2]	−1141 [−2335; −96]	0.895
NT-PRO-BNP at day1 (pg/mL)	2409 [939; 4488]	3182 [1874; 5208]	0.178
Hematocrit at day 1 (%)	29.1 [28.0; 32.4]	29.5 [26.1; 31.8]	0.469
Protein at day 1 (g/L)	60.0 [55.0; 64.0]	58.0 [54.0; 62.5]	0.550
Albumin at day 1 (g/L)	23.0 [21.0; 26.0]	23.5 [21.0; 26.0]	0.656
Change in hematocrit at day 1 (%)	−0.70 [−3.30; 0.90]	−1.35 [−2.73; −0.35]	0.251
Change in protein at day 1 (g/L)	1.00 [−2.00; 5.00]	1.00 [0.00; 3.00]	0.638
Change in albumin at day 1 (g/L)	0.00 [−1.00; 1.00]	0.00 [−2.00; 1.00]	0.992
Hemoconcentration, n (%):	18 (48.6%)	13 (32.5%)	0.226
Pseudo-WRF, n (%)	6 (16.2%)	3 (7.5%)	0.241
Mortality, n (%)	7 (18.9%)	3 (7.5%)	0.182

Data are presented as median [25th–75th percentile] or frequencies (%). *Pseudo-WRF* WRF with hemoconcentration, *True WRF* WRF without hemoconcentration

### Secondary outcomes

Both groups exhibited a similar response to loop diuretic prescription. The median loop diuretic adjusted diuresis at 2 h was 545 mL [251–1038] in the non-congestive group and 600 mL [334–858] in the congestive group (*p* = 0.950). The median natriuresis at 2 h was 104 mmol/L [68.5–122] and 93.0 mmol/L [71.5–111] in the non-congestive group and the congestive group, respectively (*p* = 0.355). The treatment resulted in similar cumulative loop diuretic prescription (120 mg [67.5–161] and 100 mg [45–140], *p* = 0.303) and fluid balance at discharge (−685 mL [−2418 to −2] and −1141 mL [−2335 to −96], *p* = 0.895).

Concerning hemoconcentration markers, no significant differences were observed between groups for individual markers (hematocrit, protein, and albumin levels). Hemoconcentration occurred at a similar frequency between the groups: 18 patients (48.6%) and 13 patients (32.5%), for non-congestive and congestive patients, respectively (p = 0.226). Pseudo-WRF also occurred at similar rates in both groups: 6 patients (16.2%) and 3 patients (7.50%) for non-congestive and congestive patients, respectively (*p* = 0.241). Results are shown in Table 2*.*

The mortality rate was 18.9% (7 patients) in the non-congestive group and 7.5% (3 patients) in the congestive group (p = 0.182).

### Multivariable analysis

Results of Firth's penalized logistic regression are shown in Table 3. After adjusting for surgical admission, VExUS ≥ 2 was not significantly associated with WRF (adjusted OR 0.54, 95% CI 0.18–1.53, *p* = 0.247), while surgical admission showed a non-significant trend toward a protective effect (adjusted OR 0.37, 95% CI 0.12–1.10, p = 0.074).

**Table 3:** Adjusted odds ratios for worsening renal function after adjustment for surgical admission using Firth's penalized logistic regression

	Odds ratio	95 CI	*p*
VExUS ≥ 2	0.54	0.18; 1.53	0.247
Surgical admission	0.37	0.12; 1.10	0.074

### Sensitivity analysis

Results of the sensitivity analysis are displayed in Table 4*.* Restricting to the 59 patients (77%) achieving a negative cumulative fluid balance. yielded consistent results with the primary analysis (WRF: 35.7% vs 19.4%, p = 0.263).

**Table 4:** Sensitivity analyses: restriction to patients with negative cumulative fluid balance

Negative fluid balance	VEXUS < 2 (n = 28)	VEXUS ≥ 2 (n = 31)	*p*
WRF, n (%)	10 (35.7%)	6 (19.4%)	0.263
Hemoconcentration, n (%):	15 (53.6%)	10 (32.3%)	0.164
Pseudo-WRF, n (%)	5 (17.9%)	2 (6.5%)	0.240

## Discussion

Our findings tend to indicate that severe congestive pattern is not associated with worsening renal function when patients are treated with loop diuretic. In addition, a high VExUS score was not associated with efficient decongestion as assessed by loop-diuretic adjusted diuresis, natriuresis, and fluid balance. Pseudo-WRF, defined by the association of WRF and hemoconcentration, and supposed to reflect efficient decongestion, did not occur differently between congestive and non-congestive patients.

One perspective could be that WRF was not higher in congestive patients because decongestion prevented further renal deterioration. In this scenario, diuretic response should have been higher in congestive patient. Nevertheless, we did not show higher diuretic response in congestive patients, and fluid balance remained similar. Conversely, another perspective suggests that the rate of WRF may actually increase, as efficient decongestive therapy could initially induce transient WRF without correlating to adverse outcomes [8, 9, 19, 20]. This assertion holds true only when patients exhibit a sufficient response to diuretic prescriptions in acute heart failure (AHF). In fact, hemoconcentration following efficient diuretic therapy may artificially increase creatinine concentration. Therefore, the fluid status of a given patient could be accurately tracked by regular assessment of hemoconcentration, which not only associates with better weight loss and diuretic response [11], but also portend favorable outcomes when persisting at discharge [21]. Therefore, we might hypothesize that the decongestion was not intensive enough in our cohort to yield similar findings. Indeed, hemoconcentration and pseudo-WRF were not more frequent in congestive patients. However, this interpretation should be considered alongside alternative explanations. First, hemoconcentration lacks a universal definition, rendering any results arguable [10]. Second, hemoconcentration might have been underestimated due to concomitant fluid administration; however, our sensitivity analysis, restricted to patients with negative fluid balance, did not support this hypothesis. Third, acute kidney injury (AKI) in the intensive care unit (ICU) often involves acute tubular necrosis [22] so that WRF can rarely be considered harmless. This pathophysiology may account for a loss of tubular reserve, which refers to the secretion capacity of the renal tubular pool [23], and might explain a poor diuretic response. In AHF patients, there is a disconnect between WRF and tubular injury [24], which explains why diuretic prescription could remain efficient and why pseudo-WRF can be observed. Therefore, our findings could reflect either insufficient decongestion intensity or a lack of correlation between VExUS and tubular reserve in critically ill patients, where multiple AKI mechanisms beyond venous congestion may predominate. If future studies are to be conducted, VExUS could be explored through the lens of pseudo-WRF, as defined by the combination of WRF, hemoconcentration and tubular markers.

Accordingly, Islas-Rodìguez et al. [25] demonstrated that decongestive therapy guided by VExUS did achieve significant decongestion, as assessed by natriuretic peptide dosage and clinical findings, but did not induce improvement in renal function in cardiorenal syndrome. In perioperative and mixed ICU settings, the association between VExUS and renal outcomes was weak [26]. In an analysis of ICU patients with acute kidney injury (AKI), the VExUS score did not demonstrate a significant association with composite outcomes related to renal events, even if it remained associated with higher odds of death [27], which was not the case in our study. These data further corroborate the idea that acute kidney injury (AKI) has a complex and multifactorial pathophysiology that cannot be fully captured by the VExUS score alone. In fact, in such situations, congestion may be more a consequence than a cause of AKI, making any hopes for renal improvement through decongestion uncertain. On the contrary, Sovetova et al. [17] studied ICU patients hospitalized for AHF and found an association of VExUS = 3 with WRF but poor natriuretic response. Those patients also showed the worst clinical outcomes, so that WRF could not be regarded as the sign of efficient decongestion, as suggested earlier. The presence of WRF and poor renal response to diuretic prescriptions may merely indicate that renal damage had already occurred. Therefore, we suggest VExUS score must be interpreted in the light of the clinical context, renal function and its potential tubular damage before expecting clinical improvement with diuretic prescription. 

Our study has several limitations. First, the observational nature of our study makes our interpretation somewhat exploratory. Second, the absence of a significant difference in our study may be attributed to insufficient statistical power. Based on effect sizes from a recent meta-analysis [28], a sample size calculation indicates that approximately 120–130 patients would be required to achieve 80% power to detect such an association. Third, this design also introduces potential selection bias, particularly as our population was predominantly composed of cardiovascular surgery patients (62%). Particularly, surgical patients were more frequent in the congestive group, introducing potential confounding. While multivariable analysis adjusted for this imbalance, the trend between surgical admission and WRF suggests that confounding by admission type may not be fully resolved. Fourth, methodological limitations include the lack of standardization in diuretic prescription, as treatment was left to the clinician's discretion. Fifth, we did not gather longitudinal data regarding VExUS, hemoconcentration and renal function and their temporal correlation. Tubular markers would have provided relevant insight, but were not retrieved during the study.

## Conclusion

In this population of critically ill patients systematically treated with loop diuretics, VExUS score was not significantly associated with worsening renal function. Similarly, VExUS did not appear to correlate with diuretic response as assessed by loop-diuretic adjusted diuresis, natriuresis, and fluid removal. Furthermore, VExUS score was not associated with hemoconcentration or pseudo-WRF following diuretic prescription. These preliminary findings suggest that larger studies may be needed to better understand the potential relationship between VExUS and renal outcomes or diuretic response in this patient population.

## Supplementary Information

Supplementary material 1.

Supplementary material 2.

Supplementary material 3.

Supplementary material 4.

## Data Availability

Data are available on reasonable request.

## Abbreviations

AKI: Acute kidney injury
AHF: Acute heart failure
BMI: Body mass index
CAD: Coronary artery disease
CI: Cardiac index
CVP: Central venous pressure
FST: Furosemide stress test
ICU: Intensive care unit
MAP: Mean arterial pressure
PAPs: Systolic pulmonary arterial pressure
VExUS: Venous excess ultrasound
SAPSII: Simplified Acute Physiologic Score II
WRF: Worsening renal function
