# Supplementary material for: Association between VExUS score and worsening renal function during diuretic therapy in the ICU
Source: Intensive Care Med Exp. 2026 Mar 31;14:40. doi: 10.1186/s40635-026-00890-9 (PMC13035985; doi:10.1186/s40635-026-00890-9)
Supplement: Supplementary file 1 — Supplementary material 1. [file 40635_2026_890_MOESM1_ESM.docx]

|  | **Normality** | **Mild congestion** | **Severe congestion** |
| --- | --- | --- | --- |
| Supra-hepatic veins | S/D ratio > 1 | S/D ratio < 1 | D wave only or S reversed |
| Portal vein doppler pulsatility | < 30% | 30-49% | ≥ 50% |
| Intra-venous doppler | Continuous pattern | Discontinuous pattern | Discontinuous pattern with Diastolic wave only |
| **VEXUS 0** | **VExUS 1** | **VExUS 2** | **VExUS 3** |
| IVC < 2 cm | IVC ≥ 2 cm | IVC ≥ 2 cm | IVC ≥ 2 cm |
|  | Normal or mild congestive pattern | One severe congestive pattern | Two severe congestive pattern at least |

**Supplementary Table 1 -** VExUS Grading System: Ultrasound Criteria for Venous Congestion Assessment. *IVC, Inferior Vena Cava.*
