# Supplementary material for: Association between VExUS score and worsening renal function during diuretic therapy in the ICU
Source: Intensive Care Med Exp. 2026 Mar 31;14:40. doi: 10.1186/s40635-026-00890-9 (PMC13035985; doi:10.1186/s40635-026-00890-9)
Supplement: Supplementary file 2 — Supplementary material 2. [file 40635_2026_890_MOESM2_ESM.docx]

|  | **Non-congestive (n=37)** | **Congestive (n=40)** | ***p*** |
| --- | --- | --- | --- |
| VEXUS rank: |  |  | <0.001 |
| 0 | 19 (51.4%) | 9 (22.5%) |  |
| 1 | 18 (48.6%) | 10 (25.0%) |  |
| 2 | 0 (0.00%) | 14 (35.0%) |  |
| 3 | 0 (0.00%) | 7 (17.5%) |  |
| E/A | 1.16 [0.96 ; 1.84] | 1.61 [1.26 ; 2.25] | 0.010 |
| E/e’ | 9.95 [7.08 ; 13.9] | 9.84 [7.50 ; 13.3] | 0.751 |
| LVEF (%) | 45.0 [35.0 ; 50.0] | 40.0 [33.8 ; 50.0] | 0.474 |
| TAPSE (mm) | 14.1 [11.3 ; 18.1] | 9.69 [8.27 ; 12.3] | <0.001 |
| PAPs (mmHg) | 43.5 (14.2) | 37.8 (10.6) | 0.093 |
| TAPSE/PAPs | 0.32 [0.21 ; 0.43] | 0.24 [0.20 ; 0.38] | 0.279 |
| IVC (cm) | 2.08 [1.78 ; 2.38] | 2.36 [2.15 ; 2.60] | 0.003 |
| Portal Pulsatility (%) | 0.25 [0.20 ; 0.35] | 0.38 [0.29 ; 0.57] | <0.001 |
| Portal Pulsatility Rank |  |  | <0.001 |
| <30% | 22 (61.1%) | 11 (27.5%) |  |
| 30-50% | 13 (36.1%) | 15 (37.5%) |  |
| >50% | 1 (2.78%) | 14 (35.0%) |  |
| Supra-hepatic vein S/D ratio | 1.08 [0.85 ; 1.40] | 0.59 [-0.59 ; 0.72] | <0.001 |
| Supra hepatic vein pattern |  |  | <0.001 |
| S/D >1 | 22 (61.1%) | 6 (15.0%) |  |
| S/D <1 | 13 (36.1%) | 17 (42.5%) |  |
| D alone, or S reversed | 1 (2.78%) | 17 (42.5%) |  |
| Intra-renal venous doppler: |  |  | 0.117 |
| Continuous | 16 (44.4%) | 14 (35.0%) |  |
| Mild | 20 (55.6%) | 24 (60.0%) |  |
| Severe | 0 (0.00%) | 2 (5.00%) |  |

**Supplementary table 2 –** Ultrasound detail according to VExUS group. The quantitative data are presented as the mean +/- standard deviation. *LVEF, Left ventricular ejection fraction; PAPs, Systolic pulmonary artery pressure; TAPSE ; Tricuspid annular plane systolic excursion*
