# Supplementary material for: Association between VExUS score and worsening renal function during diuretic therapy in the ICU
Source: Intensive Care Med Exp. 2026 Mar 31;14:40. doi: 10.1186/s40635-026-00890-9 (PMC13035985; doi:10.1186/s40635-026-00890-9)
Supplement: Supplementary file 3 — Supplementary material 3. [file 40635_2026_890_MOESM3_ESM.docx]

|  | **Intra-observer variability (%)** | **IQR** | **Intraclass Correlation Coefficient** | **CI95%** |
| --- | --- | --- | --- | --- |
| IVC | 10.5 | [4.0 ; 18.8] | 0.82 | [0.69 ; 0.91] |
| Portal Pulsatility | 10.0 | [4.0 ; 16.0] | 0.88 | [0.72 ; 0.95] |
| Supra-hepatic vein S/D ratio | 13.4 | [1.9 ; 26.5] | 0.80 | [0.70 ; 0.87] |
| Renal venous impedance index | 5.9 | [2.6 ; 10.1] | 0.73 | [0.51 ; 0.84] |

**Supplementary Table 2.** Intra-observer variability and intraclass correlation coefficient of ultrasound measurements. *IQR stands for interquartile range (25^th^ to 75^th^ percentile) and CI95% stands for 95% confidence interval.*
