# Supplementary material for: Association between VExUS score and worsening renal function during diuretic therapy in the ICU
Source: Intensive Care Med Exp. 2026 Mar 31;14:40. doi: 10.1186/s40635-026-00890-9 (PMC13035985; doi:10.1186/s40635-026-00890-9)
Supplement: Supplementary file 4 — Supplementary material 4. [file 40635_2026_890_MOESM4_ESM.docx]

|  | **Missing values frequency** |  |
| --- | --- | --- |
| ***VExUS Ultrasound markers*** |  |  |
| VExUS (baseline), n (%) | 0 (0.0%) |  |
| VExUS (2 hours), n (%) | 0 (0.0%) |  |
| VExUS (day 1), n (%) | 0 (0.0 %) |  |
| IVC (baseline), n (%) | 0 (0.0%) |  |
| IVC (2 hours), n (%) | 0 (0.0%) |  |
| IVC (day 1), n (%) | 0 (0.0%) |  |
| Portal Pulsatility (baseline), n (%) | 0 (0.0%) |  |
| Portal Pulsatility (2 hours), n (%) | 0 (0.0%) |  |
| Portal Pulsatility (day 1), n (%) | 1 (1.3%) |  |
| Supra-hepatic vein S/D ratio (baseline), n (%) | 0 (0.0%) |  |
| Supra-hepatic vein S/D ratio (2 hours), n (%) | 0 (0.0%) |  |
| Supra-hepatic vein S/D ratio (day 1), n (%) | 2 (2.6%) |  |
| Intra-renal venous doppler (baseline), n (%) | 1 (1.3%) |  |
| Intra-renal venous doppler (2 hours), n (%) | 4 (5.2%) |  |
| Intra-renal venous doppler (day 1), n (%) | 11 (14.3%) |  |
| ***Biological data*** | |  |
| Creatinine (baseline), n (%) | | 0 (0.0%) |
| Creatinine (2 hours), n (%) | | 0 (0.0%) |
| Creatinine (day 1), n (%) | | 0 (0.0%) |
| Creatinine (discharge), n (%) | | 0 (0.0%) |
| NT-PRO-BNP (baseline), n (%) | | 1 (1.3%) |
| NT-PRO-BNP (day 1), n (%) | | 0 (0.0%) |
| Natriuresis (2 hours), n (%) | | 3 (3.9%) |
| Hemoglobin (baseline), n (%) | | 0 (0%) |
| Hemoglobin (day 1), n (%) | | 0 (0%) |
| Hematocrit (baseline), n (%) | | 0 (0.0%) |
| Hematocrit (day 1), n (%) | | 0 (0.0%) |
| Protein (baseline), n (%) | | 0 (0.0%) |
| Protein (day 1), n (%) | | 0 (0.0%) |
| Albumin (baseline), n (%) | | 0 (0.0%) |
| Albumin (day 1), n (%) | | 0 (0.0%) |

**Supplementary Table 4 -** Frequency of Missing Values in Study Population.
